# Correction to “Injectable Genetic Engineering Hydrogel for Promoting Spatial Tolerance of Transplanted Kidney in Situ”

**DOI:** 10.1002/advs.74915

**Published:** 2026-03-20

[J. Lin, S. Liu, X. Xue, J. Lv, L. Zhao, L. Yu, H. Wang, J. Chen, Injectable Genetic Engineering Hydrogel for Promoting Spatial Tolerance of Transplanted Kidney in Situ. Adv. Sci. 2024, 11, 2408631. https://doi.org/10.1002/advs.202408631]

The authors identified error in the preparation of the rheology figures: the panels were inadvertently formatted using an incorrect plotting template. The rheology panels in Figure 1G and Figures S4 and S5A,C have been reprocessed directly from the native instrument, with all measured data points displayed. These changes do not alter the results or conclusions of the article. The correct image should be as follows:

## Experimental Section

Rheological Characterization: Hydrogel rheology was characterized using a TA Instruments DHR‐2 hybrid rheometer equipped with a Peltier temperature control stage. Samples were loaded onto a 20 mm titanium parallel plate geometry (P20 TiL) with a 0.5 mm gap and tested at 37°C (unless otherwise specified). Oscillatory measurements included an amplitude sweep at 5 Hz (0.01%–10 000% strain) to identify the LVE regime and yielding behavior, followed by a frequency sweep performed within the LVE regime at 1% strain over 0.01–600 rad s^−1^. For steady shear characterization, the shear rate was ramped from 0.01 to 8000 s^−1^, and the apparent viscosity was recorded as a function of shear rate. Time‐dependent shear responses were assessed using a step‐shear protocol consisting of 100 s^−1^ for 35 s followed by 1 s^−1^ for 60 s. All data were acquired and analyzed using “TA Instruments TRIOS/Orchestrator software.”


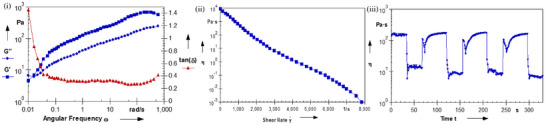


**FIGURE 1G**. Rheological characterization of iGE‐Gel. (i) After selecting 1% (within the LVE range) by oscillatory strain sweep, a frequency sweep was performed to show storage modulus (G′), loss modulus (G″), and tan δ (G″/G′). (ii) Steady‐shear viscosity measured over a shear‐rate ramp. (iii) Time‐dependent viscosity measured under an alternating low/high shear protocol.


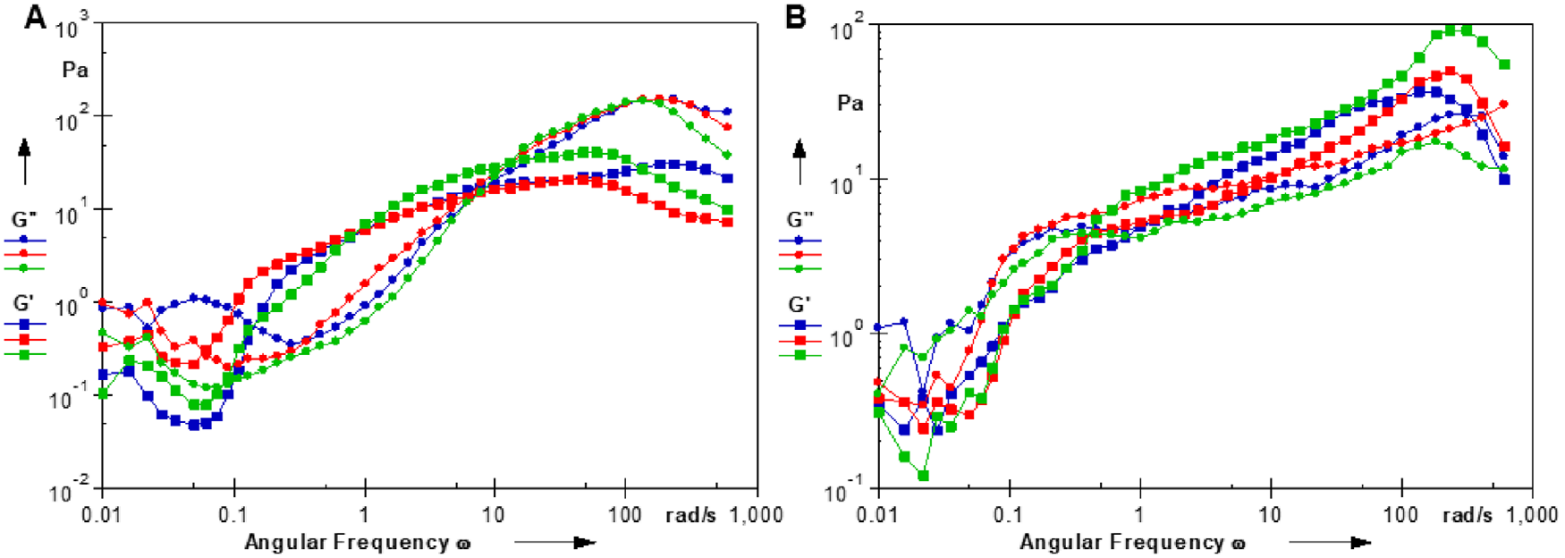


**FIGURE S4**. Oscillatory frequency sweep of the HA and Foe‐EVs (A) and HASA alone (B).


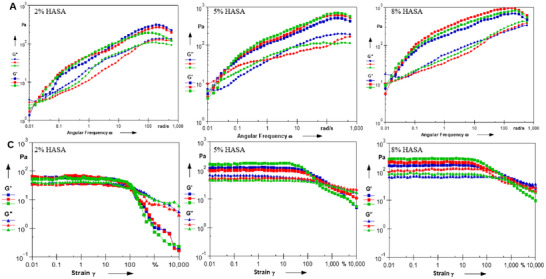


**FIGURE S5**. (A) Oscillatory frequency sweep of iGE‐Gel comprising serial HASA and 1 × 10^14^ particles of engineered EVs. (C) Oscillatory strain sweep of iGE‐Gel comprising serial HASA and 1 × 10^14^ particles of engineered EVs.

Meanwhile, rheology measurements at different temperatures (25°C, 32°C, and 42°C) were provided in Figure S18. With increasing temperature from 25°C to 42°C, both G′ and G″ decreased monotonically while G′ remained higher than G″ across all temperatures, indicating an elastic‐dominant gel state (Figure S18A,B). Notably, the mean yield strain dropped sharply with temperature, decreasing from 3643.75% at 25°C to 495.66% at 42°C, suggesting markedly reduced deformation tolerance and earlier network yielding at elevated temperatures (Figure S18C,D).


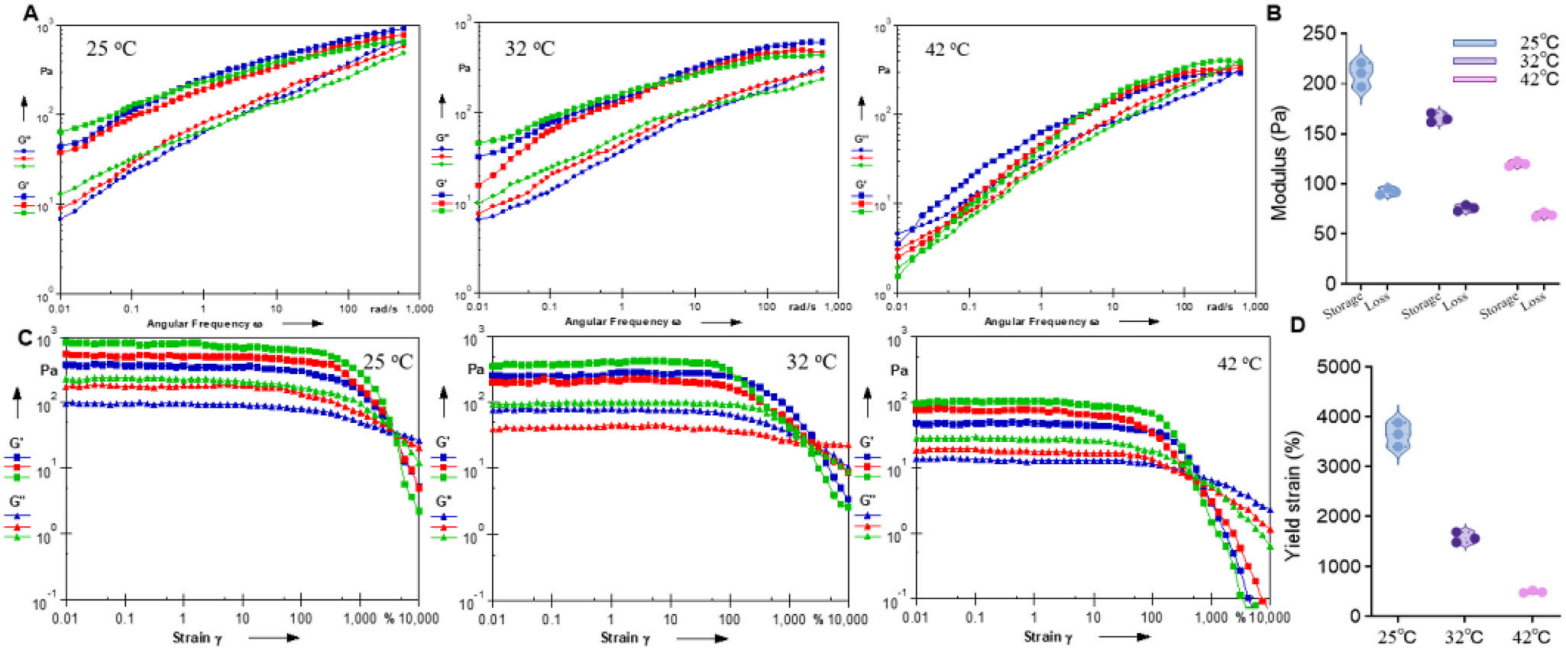


**FIGURE S18**. (A) Oscillatory frequency sweep of iGE‐Gel measured at 25°C, 32°C, and 42°C at a fixed strain of 1% (within the LVE range). (B) Storage and loss modulus at ω = 10 rad/s at different temperatures. (C) Oscillatory strain sweep of iGE‐Gel measured at 25°C, 32°C, and 42°C at a fixed frequency of 5 Hz. (D) Yield strain at different temperatures, defined as the G′–G″ crossover.

The authors identified that a key prior work was not sufficiently cited. We have therefore added the appropriate citation in both the Introduction and Methods at the relevant statements, as follows:

“Introduction: …Injectable phospholipid vesicle crosslinked hydrogels for programmable delivery and their rheological characterization have been reported previously [18]. Our work builds upon these concepts by integrating engineered natural EVs with HASA to form an immunomodulatory iGE‐Gel for in situ spatial tolerance in kidney transplantation…”

“*iGE‐Gel formation: …*are loaded individually into distinct 1‐mL luer lock syringes and subsequently securely interconnected through a luer lock elbow connector, following an established syringe‐coupling approach [18].”

[18] Correa S, Grosskopf AK, Klich JH, Hernandez HL, Appel EA. Injectable Liposome‐based Supramolecular Hydrogels for the Programmable Release of Multiple Protein Drugs. Matter. 2022 Jun 1;5(6):1816‐1838.

We apologize for this error.

